# The expression characteristics and prognostic roles of autophagy-related genes in gastric cancer

**DOI:** 10.7717/peerj.10814

**Published:** 2021-02-03

**Authors:** Mengya Wang, Jingjing Jing, Hao Li, Jingwei Liu, Yuan Yuan, Liping Sun

**Affiliations:** 1 Tumor Etiology and Screening Department of Cancer Institute, and Key Laboratory of Cancer Etiology and Prevention in Liaoning Education Department, the First Hospital of China Medical University, Shenyang, China; 2 Key Laboratory of GI Cancer Etiology and Prevention in Liaoning Province, the First Hospital of China Medical University, Shenyang, China; 3 Department of Clinical Laboratory, the First Hospital of China Medical University, Shenyang, China

**Keywords:** Autophagy, Gastric cancer, Gene expression, TCGA, *ATG4*

## Abstract

**Background:**

Autophagy is an evolutionally highly conserved process, accompanied by the dynamic changes of various molecules, which is necessary for the orderly degradation and recycling of cellular components. The aim of the study was to identify the role of autophagy-related (*ATG*) genes in the occurrence and development of gastric cancer (GC).

**Methods:**

Data from Oncomine dataset was used for the differential expression analysis between cancer and normal tissues. The association of *ATG* genes expression with clinicopathologic indicators was evaluated by The Cancer Genome Atlas (TCGA) database and Gene Expression Omnibus (GEO) database. Moreover, using the TCGA datasets, the prognostic role of *ATG* genes was assessed. A nomogram was further built to assess the independent prognostic factors.

**Results:**

The expression of autophagy-related genes *AMBRA1*, *ATG4B*, *ATG7*, *ATG10*, *ATG12*, *ATG16L2*, *GABARAPL2*, *GABARAPL1*, *ULK4* and *WIPI2* showed differences between cancer and normal tissues. After verification, *ATG14* and *ATG4D* were significantly associated with TNM stage. *ATG9A*,* ATG2A*, and *ATG4D* were associated with T stage. *VMP1* and *ATG4A* were low-expressed in patients without lymph node metastasis. No gene in autophagy pathway was associated with M stage. Further multivariate analysis suggested that *ATG4D* and *MAP1LC3C* were independent prognostic factors for GC. The C-index of nomogram was 0.676 and the 95% CI was 0.628 to 0.724.

**Conclusion:**

Our study provided a comprehensive illustration of *ATG* genes expression characteristics in GC. Abnormal expressions of the ubiquitin-like conjugated system in *ATG* genes plays a key role in the occurrence of GC. *ATG8/LC3* sub-system may play an important role in development and clinical outcome of GC. In the future, it is necessary to further elucidate the alterations of specific *ATG8/LC3* forms in order to provide insights for the discovery, diagnosis, or targeting for GC.

## Introduction

Autophagy is an evolutionally highly conserved process, which is necessary for the orderly degradation and recycling of cellular components (Yang et al., 2020). In normal cells, autophagy keeps low-level constitutive function. Basal autophagy plays an important role in maintaining homeostatic control and elimination of unfavorable proteins. Its activity can be accelerated by a variety of cellular stressors including nutrient starvation, DNA damage, and organelle damage. Autophagy is closely related to the occurrence and treatment of tumors (Rahman et al., 2020). Recently, the paradoxical roles of autophagy in tumor suppression and tumor promotion have been widely observed. As a physiological quality control process, autophagy exerts a cytoprotective effect to suppress cancer development by removing damage that leads to aberrant mutations. On the other hand, as cancer progresses, starving and oxidative stress situation can active autophagy to fulfill the high metabolic need of cancer cells (Mathew, Karantza-Wadsworth & White, 2007).

The process of autophagy is accompanied by the dynamic changes of various molecules. Identification of the autophagy-related biomarkers will contribute to improving diagnosis and treatment of cancers. Autophagy is executed by a set of autophagy-related (*ATG*) genes, which have been investigated extensively in yeast. Although the discovery of *ATG* genes greatly advanced the understanding of autophagy, the function and mechanisms involved in *ATG* genes need to be further explored in mammalian. Recently, several studies have investigated the association of *ATG* genes and cancers. By activating *ATG6*-mediated autophagy, the down-regulation of microRNA-30a increases the chemoresistance of osteosarcoma cells, thereby inhibiting cell proliferation and invasion (Xu et al., 2016). Upregulation of UCA1 inhibits cell proliferation, migration, invasion, and drug resistance via *ATG7*-mediated autophagy (Wu et al., 2019). The methyltransferase MGMT inhibits the expression of *ATG4B*, thereby inhibiting autophagy and reducing the chemosensitivity of cisplatin in gastric cancer (GC) (Lei et al., 2020). Moreover, comprehensive study of all *ATG* genes has been conducted in breast, head neck and kidney carcinoma (Deng et al., 2018; Pei et al., 2018).

GC is the fourth most common cancer and the second leading cause of cancer death in the world (Van Cutsem et al., 2016). The incidence is mainly related to diet, lifestyle, genetic predisposition, family history, treatment and medical conditions, infections, demographic characteristics, occupational exposures and ionizing radiation (Yusefi et al., 2018). Abnormal expression of *ATG* gen*es* may lead to the dysregulation of autophagy and tumorigenesis. However, the diagnostic and prognostic values of *ATG* genes have not been fully realized in GC. Since large-scale expression data is available, it is feasible to display an overview of *ATG* genes from the perspective of expression characteristics and prognostic role in GC. In the current study, we performed systematic analysis by using available datasets of ONCOMINE and The Cancer Genome Atlas (TCGA), in order to evaluate the differential expression of *ATG* genes and their associations with clinicopathological parameters and prognosis of GC. Our data may provide a new understanding of the autophagy-related mechanism in gastric carcinogenesis.

## Materials & Methods

### Autophagy-related genes selection

**Figure 1:**
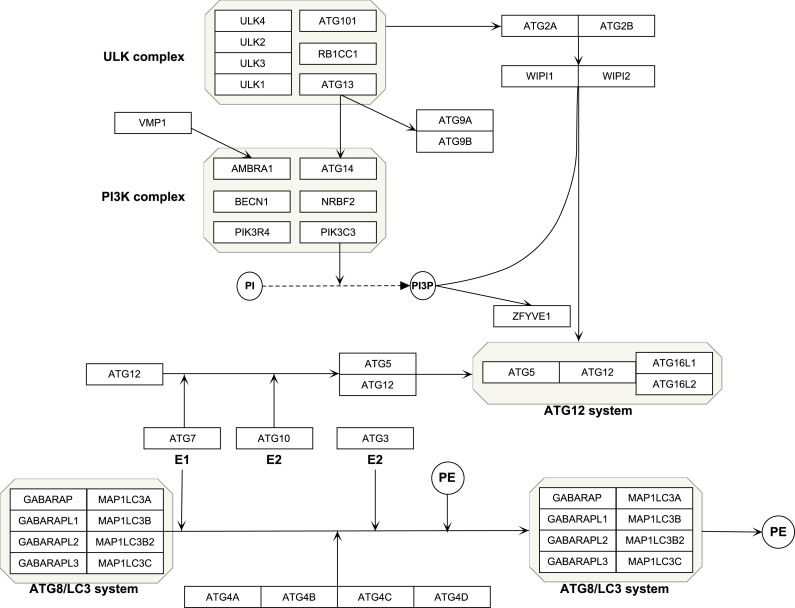
Schematic of autophagy pathway.

The Kyoto Encyclopedia of Genes and Genomes (KEGG, https://www.kegg.jp/) is an online tool for analysis of the gene function (Kanehisa et al., 2020). Reactome (https://reactome.org/) is a bioinformatics resource for visualization, interpretation and analysis of pathways (Jassal et al., 2020). Using the two datasets, we selected the genes in autophagy pathways as *ATG* genes, which composed four functional units including the *ULK* protein complex, *Beclin-1/PI3K* complex, ubiquitin-like conjugation system and other genes (Mizushima, Yoshimori & Ohsumi, 2011). All the isoforms of a gene were included, such as *ATG4A*, *ATG4B*, *ATG4C* and *ATG4D*. A total of 40 genes were selected. PathVisio (Version:3.3.0, https://pathvisio.github.io/) was used to visualize the autophagy genes (Kutmon et al., 2015). which composed four functional units including *ULK* complex, *PI3K* complex, ubiquitin-like conjugation system and other genes (Table 1 and Fig. 1).

**Table 1:** Description of autophagy related gene.

	Gene symbol	Aliases	Function
ULK complex	ULK1/2/3/4	ATG1A/B/C/D	Acts upstream of PIK3C3 to regulate the formation of autophagophores
	ATG101	C12orf44	Stabilizes ATG13, protecting it from proteasomal degradation.
	ATG13	KIAA0652	Essential for autophagosome formation
	RB1CC1	ATG17	Direct interaction with Atg16L1
PI3K complex	BECN1	ATG6	Acts as core subunit of the PI3K complex
	PIK3R4	VPS15	Involved in regulation of degradative endocytic trafficking
	PIK3C3	VPS34	Catalytic subunit of the PI3K complex
	NRBF2	COPR	Modulated ATG14 protein
	ATG14	ATG14L	Plays a role in autophagosome formation and MAP1LC3/LC3 conjugation to phosphatidylethanolamine
	AMBRA1	DCAF3	Interacts with becn1
ubiquitin-like conjugating system	ATG12	APG12	Conjugation with ATG5
	ATG5	APG5	Functions as an E1-like activating enzyme
	ATG16L1/L2	ATG16A/B	Interacts with ATG12-ATG5 to mediate the conjugation of phosphatidylethanolamine (PE) to LC3
	ATG3	APG3	E2 conjugating enzyme
	ATG4A/B/C/D	APG4A/B/C/D	Cleaves the C-terminal amino acid of ATG8 family proteins to reveal a C-terminal glycine
	ATG7	APG7	E1-like activating enzyme
	ATG10	APG10	E2-like enzyme
	GABARAP/L1/L2/L3	ATG8A/B/C/D	Ubiquitin-like modifier
	MAP1LC3A/B/B2/C	ATG8E/F/G/J	Ubiquitin-like modifier
others	WIPI1/2	ATG18A/B	Functions upstream of the ATG12-ATG5-ATG16L1 complex and LC3, and downstream of the ULK1 and PI3-kinase complexes
	ATG9A/B	APG9L1/L2	Transmembrane protein
	ATG2A/B	/	Required for both autophagosome formation
	ZFYVE1	DFCP1	PI3P-binding FYVE-containing protein
	VMP1	EPG3, TANGO5, TMEM49	Plays a role in the initial stages of the autophagic process through its interaction with BECN1

### Differential gene expression analysis by Oncomine

By consulting the public data in Oncomine (https://www.oncomine.org/resource/login.html) (Rhodes et al., 2004), a powerful online database with 715 sub datasets and 86,733 samples, we established and logged in an Oncomine account, and input all of 40 *ATG* genes (gene symbols were listed in Table 1) to analyze their differential expression at transcription level in GC and different Lauren types. Combination of *P*-value <0.05 and fold change >2 was identified as significant difference in gene expression.

### Correlation analysis of *ATG* genes expression and clinicopathological parameters from TCGA and GEO datasets

TCGA is a public database that contains the data of genomic expressions and the clinical features in 33 types of cancers (Tomczak, Czerwinska & Wiznerowicz, 2015). The gene expression and clinicopathological information of GC were downloaded from TCGA data portal (https://portal.gdc.cancer.gov/projects/). R was performed to normalize the expression data. The patients’ TNM stage, T, N and M (Nagtegaal et al., 2020) were considered as the clinical parameters.

### Verification of the differences of gene expression

The GSE62254 dataset was a 300 samples microarray profile tested by the Asian Cancer Research Group (ACRG) (Cristescu et al., 2015). Using GSE62254, we verified the differences of gene expression identified from TCGA datasets.

### Statistical analysis

All statistical analyses were performed by R 3.14 (http://www.r-project.org/) and the package of rms. Student’s *t*-tests was used to analyze the differences between cancer samples and normal tissues, of which the criterion is *p*-value <0.01 and fold change >2.0. The association between the *ATG* genes expressions and clinical features was accessed by Pearson X^2^ test. The correlation between *ATG* genes expressions and overall survival time was evaluated by Kaplan–Meier method and compared by log-rank test. Univariate and multivariate Cox proportional hazard regression models were used to recognize the independent prognostic factors. Based on the multivariate Cox regression models, a nomogram was formulated together with all the independent prognostic genes. The concordance index (C-index), which is similar to the area under the receiver operating characteristic (ROC), was used to evaluate the nomogram. *P* < 0.05 were considered significant difference.

## Results

### Differential expression of *ATG* genes in GC

**Figure 2:**
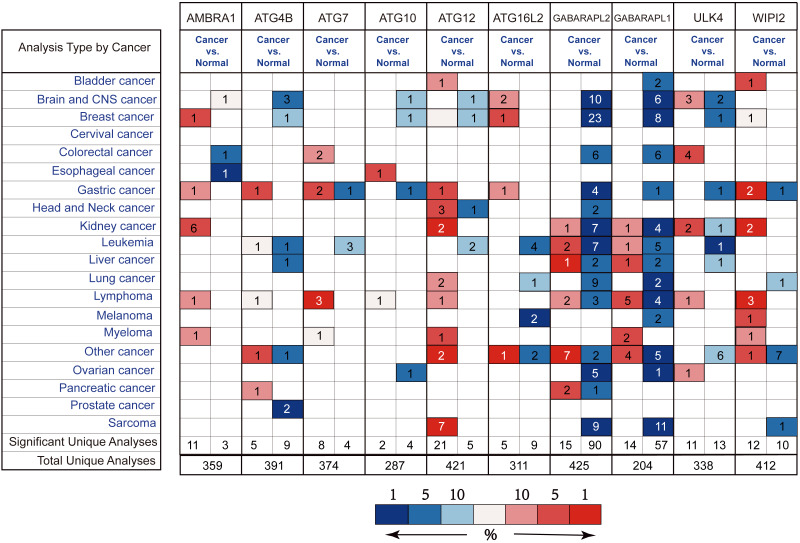
Different ATGs mRNA expression in different tumor types. This graphic showed the numbers of datasets with statistically signifcant mRNA overexpression (red) or downexpression (blue) of the target gene (cancer vs. normal tissue). Cell color is determined by the best gene rank percentile for the analyses within the cell.

By the Oncomine analysis, there were 10 genes of 40 *ATG* genes with significantly differential expression between GC and normal samples, which were named as differentially expressed genes (DEGs) (Fig. 2). Seven DEGs were belong to the ubiquitin-like conjugating system, among them *ATG4B*, *ATG12* and *ATG16L2* were significantly up-regulated in GC, while *ATG10*, *GABARAPL2* and *GABARAPL1* expressions were down-regulated in GC. As for *ATG7*, the expression was uncertain. *ULK4*, belonging to the *ULK* complex, was found down-regulated in GC. While *AMBRA1*, a member of the *PI3K* complex, was highly expressed in GC. As a connection between *PI3K* complex, *ULK* complex and *ATG12* system, *WIPI2* showed higher expression in cancer tissue.

**Figure 3:**
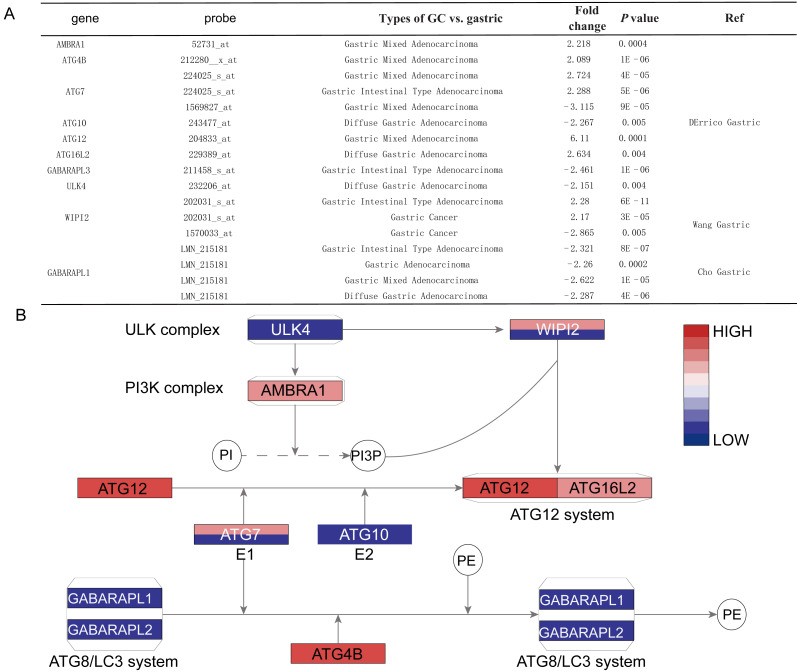
The detail information for the different ATGs. (A) The detail information in the oncomine dataset. (B) The position of different ATGs in autophagy pathway. The blue color represents downexpression in cancer, while the red color represents overexpression in cancer. The gene with two different colors means two probe of the gene showed different expressions. The gradient color represents the gene’s fold change.

Histological stratification analysis showed that *GABARAPL1* was down-regulated in all types of GC compared with normal tissues, with fold change of −2.321 in intestinal gastric adenocarcinoma, −2.287 fold in diffuse adenocarcinoma and −2.622 fold in mixed adenocarcinoma. Six DEGs showed significant differences in the gastric mixed adenocarcinoma subgroup, among them *AMBRA1*, *ATG4B*, *ATG7* (probe 224025_s_at) and *ATG12* were up-regulated, while *GABARAPL1* and *ATG7* (probe 1569827_at) were down-regulated. Four DEGs including *ATG10*, *ATG16L2*, *ULK4* and *GABARAPL1* showed differences in diffuse gastric adenocarcinoma subgroup, while other four DEGs including *ATG7* (probe 224025_s_at), *GABARAPL1*, *WIPI2* and *GABARAPL3* showed differences in gastric intestinal type adenocarcinoma subgroup (Figs. 3A and 3B).

### Association between *ATG* genes expression and clinicopathologic variables of GC

Data of 376 GC patients in TCGA were downloaded for the analysis. *ATG14*, *ULK3*, *ATG2B*, *ATG12*, *ATG4C*, *ATG4D*, and *MAP1LC3A* showed significantly relationship with TNM stage. After verification, *ATG14* and *ATG4D* were significantly associated with TNM stage (*P* = 0.027, 0.048 respectively). *ATG9A* (*P* = 0.00083), *ATG2A* (*P* = 0.00417), and *ATG4D* (*P* = 0.00864) were related with T stage. Low expression of *VMP1* and *ATG4A* suggested absence of lymph node metastasis (*P* = 0.0018, 0.015, correspondingly). However, no gene in autophagy pathway was observed to be associated with M stage (Table 2).

### Roles of *ATG* genes expression in the prediction of GC prognosis

**Figure 4:**
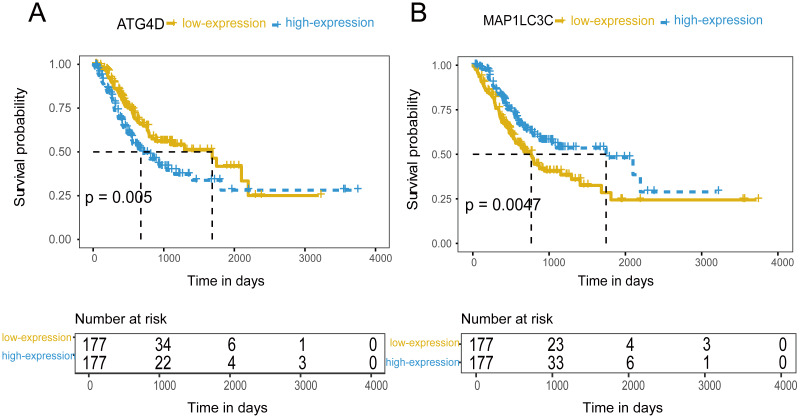
The prognostic value of mRNA level of independent prognostic factors. (A) ATG4D. (B) MAP1LC3C.

**Figure 5:**
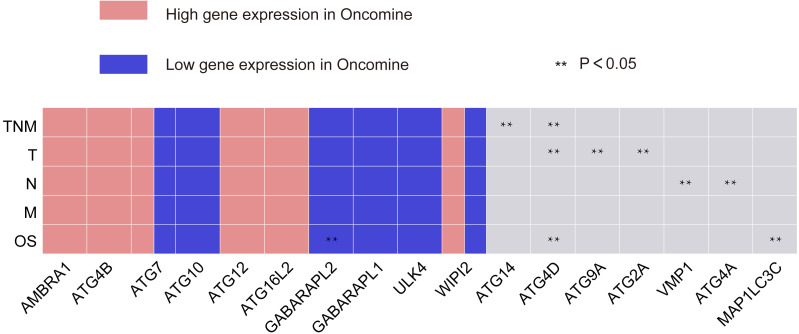
Summary of the correlation between *ATG* genes expression and TNM staging and prognosis. The red frame represents genes with high significant expression, and the blue frame represents genes with low significant expression. Two asterisks (**) represent that gene expressions has significant correlation with TNM staging or OS of GC.

354 patients were included to analyze the overall survival of GC. The median value was used as cut-off value to distinguish high expression and low expression of *ATG* genes. According to the univariate survival analysis, *ATG4D*, *GABARAPL2* and *MAP1LC3C* were significantly associated with the prognosis of GC. Moreover, the patients with low-expression of *ATG4D* or high-expression of *GABARAPL2* and *MAP1LC3C* demonstrated longer survival time, and both of the latter two genes belonged to *ATG8/LC3* system. Using the Cox’s proportional hazards model, we then performed the multivariate analysis adjusted by gender, age, TNM stage. *ATG4D* and *MAP1LC3C* were identified as the independent prognostic factors, with adjusted hazard ratio (HR) of 1.5727 (95% CI [1.1194–2.21]) and 0.5767 (95% CI [0.4086–0.8138]) separately (Fig. 4 and Table 3). The summary of the correlation between *ATG* genes expression and TNM staging and prognosis of GC was shown in Fig. 5.

### Joint prediction of the GC prognosis using *ATG4D* and *MAP1LC3C*

**Figure 6:**
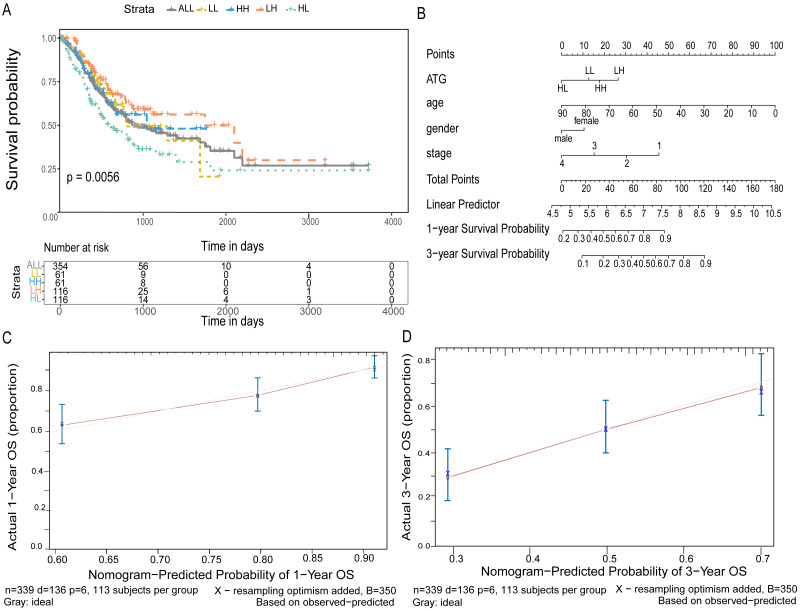
Joint predictive the patients prognosis using ATG4D and MAP1LC3C. (A) The Kaplan–Meier plot for the four groups. (B) The nomogram for indicating the one- and three-year overall survival in patients with gastric cancer. (C) The calibration curve of nomogram for predicting overall survival at one year. (D) The calibration curve of nomogram for predicting overall survival at three year.

According to the expression of *ATG4D* and *MAP1LC3C* in GC, the gastric cancer patients were divided into four groups: *ATG4D* high expression - *MAP1LC3C* high expression (HH), *ATG4D* low expression - *MAP1LC3C* low expression (LL), *ATG4D* high expression - *MAP1LC3C* low expression (HL) and *ATG4D* low expression - *MAP1LC3C* high expression (LH). A significant difference was displayed among the four groups (*p* = 0.0056, Fig. 6A).

**Table 2:** The association between autophagy related gene and TNM stage.

Gene symbol			TCGA			GSE62254		
		**TNM**					
		I–II	III–IV	*P*	I–II	III–IV	*P*
ATG14	low	96	80		72	76	
	high	71	105	0.00761	54	96	**0.02711**
ULK3	low	71	100		61	89	
	high	96	85	0.03	65	83	0.568
ATG2B	low	95	85		62	86	
	high	72	100	0.04032	64	86	0.8923
ATG12	low	96	83		58	91	
	high	71	102	0.018	68	81	0.241
ATG4C	low	94	84		65	83	
	high	73	101	0.041	61	89	0.57
ATG4D	low	70	100		55	95	
	high	97	85	0.022	71	77	**0.04822**
MAP1LC3A	low	71	105		65	83	
	high	96	80	0.0076	61	89	0.57
		**T**					
		T1T2	T3T4	P	T1T2	T3T4	P
WIPI1	low	41	147		101	49	
	high	58	121	0.022	87	63	0.0947
ATG9A	low	39	143		80	70	
	high	60	125	0.018	108	42	**0.00083**
ATG2B	low	42	145		95	85	
	high	57	123	0.047	93	57	0.092
ATG2A	low	40	141		82	68	
	high	59	127	0.038	106	44	**0.00417**
ATG4D	low	39	141		83	67	
	high	60	127	0.025	105	45	**0.00864**
ATG7	low	40	146		90	60	
	high	59	122	0.017	98	52	0.3396
		**N**					
		N0	!N0	P	N0	!N0	P
PIK3R4	low	66	116		18	132	
	high	45	130	0.031	20	130	0.728
VMP1	low	65	116		28	122	
	high	46	130	0.046	10	140	**0.00178**
ATG12	low	66	113		16	134	
	high	45	133	0.018	22	128	0.2976
ATG4A	low	68	115		26	124	
	high	43	131	0.0111	12	138	**0.01509**
		**M**					
		M0	M1	P	M0	M1	P
ULK4	low	160	19		137	13	
	high	170	6	0.008	136	14	0.8401
MAP1LC3B	low	174	7		139	11	
	high	156	18	0.0171	134	16	0.3131

**Notes.**

Significant results are marked in bold.

Furtherly, to predict 1-year and 3-year survival rate, we built a nomogram by the multivariate Cox regression models. After validation, the C-index was 0.676 and the 95% CI was 0.628 to 0.724. According to the total score after added with points identified on the point scale, we found that the likelihood of 1-year and 3-year OS for individual patient could be reasonably predicated by nomogram (Fig. 6B). As shown in Figs. 6C and 6D, the survival evaluated by the Kaplan–Meier method was marked on the y-axes, the predicted survival estimated by nomogram was observed on the x-axes, and the red lines represented the ideal reference line for which predicted survival corresponds with actual survival. The plot for the probability of OS 1-year or 3-year showed optimal agreement between the prediction by nomogram and actual observation for nomogram.

## Discussion

Considering the vital function of *ATG* genes in autophagy, many studies have focused on their role in cancers. To date, no researcher has elaborated an overview of the impact of *ATG* genes on the development, progression, and prognosis of GC. In the current study, for the first time, we investigated the expression profiling and the prognostic roles of whole members of *ATG* genes in GC using multiple databases. Our results elucidated that abnormal expressions of some key *ATG* genes were significantly associated with GC progression and outcome.

**Table 3:** Prognosis analysis of autophagy related gene in TCGA datasets.

	Univirable analysis		Multivanable analysis	
	HR(95CI)	*P*	HR(95CI)	*P*
ATG4D	1.602(1.153–2.225)	**0.00493**	1.5727(1.1194–2.21)	**0.009058**
GABARAPL2	0.6925(0.499–0.9609)	**0.0279**	0.7855(0.5597–1.102)	0.162447
MAP1LC3C	0.6242(0.4488–0.8682)	**0.00511**	0.5767(0.4086–0.8138)	**0.00173**

**Notes.**

Significant results are marked in bold.

Firstly, 10 DEGs were identified between cancer and normal tissues, and 7 of these genes were associated with ubiquitin-like conjugating system, which intimately involved in driving the biogenesis of the autophagosomal membrane (Nakatogawa, 2013). *ATG4B* (Liu et al., 2014), the core autophagy protein in *ATG8/LC3* system, was found to be up-regulated in cancer tissue in our study. It has been reported that *ATG4B* can promote the growth of colorectal cancer, while silencing the expression of *ATG4B* can reduce the colony formation of cancer cells and inhibit tumor growth (Liu et al., 2014; Liu et al., 2018). The E1-like activating enzyme *ATG7* and the E2-like activating enzyme *ATG10* also play a vital role in activating and transferring key proteins in the sub-systems. In our study, expression of *ATG12* and *ATG7* showed up-regulation while *ATG10* expression was down regulated in cancer tissues. Similarly, Cao et al. (2016) analyzed 352 tissue microarrays containing cancer and paired adjacent normal tissues and found that *ATG7*, *ATG12* were highly expressed in the GC tissues, and *ATG10* was weakly expressed in GC. Probably because autophagy plays a specific function as a cancer suppressor or tumor promoter, mainly depends on the environment, and its activity will change with the development of the tumor (Amirfallah et al., 2019). As for *ATG* genes of other functional units of ubiquitin-like conjugating system, some studies (Lebovitz et al., 2015; Su et al., 2019) found that *GABARAPL1* transcripts were less abundant in breast, prostate, liver and non-small cell lung cancers than matched normal controls, indicating that *GABARAPL1* may be a tumor suppressor. While *ATG16L2* transcripts increased in kidney cancer. As a high risk gene, its high expression is associated with poor prognosis (Wan et al., 2019). The high expression of Ambra1 is beneficial to cell survival (Sun et al., 2018). Falasca compared 26 prostate adenocarcinoma and 12 normal specimens by immunohistochemistry and observed that *AMBRA1* was highly expressed in prostate cancer (Falasca et al., 2015). The expression trend of those genes was consistent with our results in GC. The above results indicate that the ubiquitin-like conjugated system plays a key role in the occurrence of GC, and its mechanism deserves further study.

It has been reported that autophagy was associated with the invasion, migration as well as implantation metastasis of cancer. Therefore, we further analyzed the relationship between *ATG* genes and GC TNM staging, and verified the differential genes using GSE62254 to improve the accuracy. After verification, *ATG9A*, *ATG2A* and *ATG4D* were found to be associated with T stage. Among these genes, *ATG9A* was previously reported to be associated with T stage in non-metastatic renal cell carcinoma (Tang et al., 2013). *ATG4D* affects the biological behavior of prostate cancer by regulating the activity of androgen receptor (Hu et al., 2020). Besides, all of these significant differences were observed at early T stage, which suggested that autophagy may play its role mainly at the early stage of GC progression. By analyzing the expression of *ATG* genes both in TCGA and GSE62254, the results showed that *VMP1* and *ATG4A* were over-expressed in patients with lymph node metastasis. Similarly, Yang et al. found that the expression of *ATG4A* was associated with lymph node metastasis in 110 GC patients (Yang et al., 2016). *VMP1* was reported to promote Kras G12D-mediated pancreatic cancer initiation and facilitate lymph node metastasis (Loncle et al., 2016). In addition, *ATG4D* and *ATG14* were observed to be associated with overall TNM stage according to our analysis. *ATG14* was up-regulated while *ATG4D* was down-regulated in GC of stage III-IV, which suggested that the two genes may have the opposite effect in GC progression. It has been reported that the low expression of *ATG4D* was associated with Colorectal Cancer of III stage (Gil et al., 2018). Interestingly, significant relation was observed between *ATG4* isoforms and all the three clinicopathologic variables, that *ATG4D* was associated with TNM and T stage, and *ATG4A* showed difference in N stage. As *ATG4* activity is essential and highly specific to autophagy, it may be a prospective autophagy-specific target for GC therapy.

Previous investigations have also indicated the predictive role of autophagy pathway genes in prognosis of various types of cancers. Here we analyzed all the *ATG* genes using TCGA dataset to assess their prognostic values in GC. *ATG4D* and *MAP1LC3C* were confirmed to be statistically significant in multivariate survival analysis. The expression of *ATG4D* and *MAP1LC3C* is low in colorectal cancer, and *ATG4D* is related to the poor prognosis of pancreatic cancer (Hu et al., 2020). The high expression of *ATG4D* and the low expression of *MAP1LC3C* may indicate the poor survival of gastric patients. Furthermore, we developed a nomogram according to the joint expression of *ATG4D* and *MAP1LC3C* along with other clinicopathological parameters. The group of HL showed poor survival while the group of LH indicated favorable prognosis. In the internal validation set, the calibration plot showed that the predicted 3-year and 5-year overall survival were in correspondence with the actual survival estimated by the Kaplan–Meier method. *MAP1LC3C* is a member of the LC3 family of proteins and a key structural component of the autophagosome that undergoes processing by members of the *ATG4* family (Costa et al., 2016). These two functionally related genes together may have synergistic effect in GC prognosis. For the first time, our study formulated an *ATG*-based nomogram that could predict outcome of GC with a better accuracy.

On the basis of the above results, we found that *ATG4* and *ATG8*, members of *ATG8/LC3* system, were associated with both the occurrence and prognosis of GC in our study. *ATG4* was up-regulated in cancer and was associated with poor GC survival. The over-expression of *ATG8* was observed in normal tissues and involved with favorable prognosis of GC. *ATG8/LC3* is essential for autophagosome biogenesis and it also functions as an adaptor protein for selective autophagy (Lee & Lee, 2016). At the same time, it is also widely used as a marker of autophagic vacuoles (Mareninova et al., 2020).Dysregulation of *ATG8/LC3* proteins may contribute to pathogenic effects during progression of autophagy-associated human diseases. Our results indicated that the *ATG8/LC3* system may play an important role in development and clinical outcome of GC. Elucidation of alterations in specific *ATG8/LC3* forms in GC could provide insights for the discovery, diagnosis, or targeting of this high-mortality disease.

In conclusion, our study provided a comprehensive illustration of *ATG* genes expression characteristics in GC. Abnormal expressions of *ATG* genes were observed to be significantly involved in the whole process of GC occurrence, progression and prognosis. Specially, the *ULK* system, such as *ATG4* family and *ATG8/LC3*, may serve as valuable biomarkers to indicate gastric carcinogenesis and prognosis. Considering the underlying important roles of *ATG* genes in gastric carcinogenesis and progression, future molecular experiments concerning the functions and mechanisms of *ATG* genes may generate promising significance in GC development and treatment.

## Conclusions

Our study provided a comprehensive illustration of *ATG* genes expression characteristics in GC. Abnormal expressions of the ubiquitin-like conjugated system in *ATG* genes plays a key role in the occurrence of GC. *ATG8/LC3* sub-system may play an important role in development and clinical outcome of GC. In the future, it is necessary to further elucidate the alterations of specific *ATG8/LC3* forms in order to provide insights for the discovery, diagnosis, or targeting for GC.

## Additional Information and Declarations

### Data Availability

The following information was supplied regarding data availability: Data is available at Oncomine: https://www.oncomine.org/resource/login.html. Data is also available at TCGA: TCGA-STAD and at NCBI GEO: GSE62254.

## References

[ref-1] Amirfallah A, Arason A, Einarsson H, Gudmundsdottir ET, Freysteinsdottir ES, Olafsdottir KA, Johannsson OT, Agnarsson BA, Barkardottir RB, Reynisdottir I (2019). High expression of the vacuole membrane protein 1 (VMP1) is a potential marker of poor prognosis in HER2 positive breast cancer. PLOS ONE.

[ref-2] Cao QH, Liu F, Yang ZL, Fu XH, Yang ZH, Liu Q, Wang L, Wan XB, Fan XJ (2016). Prognostic value of autophagy related proteins ULK1, Beclin 1, ATG3, ATG5, ATG7, ATG9, ATG10, ATG12, LC3B and p62/SQSTM1 in gastric cancer. American Journal of Translational Research.

[ref-3] Costa JR, Prak K, Aldous S, Gewinner CA, Ketteler R (2016). Autophagy gene expression profiling identifies a defective microtubule-associated protein light chain 3A mutant in cancer. Oncotarget.

[ref-4] Cristescu R, Lee J, Nebozhyn M, Kim KM, Ting JC, Wong SS, Liu J, Yue YG, Wang J, Yu K, Ye XS, Do IG, Liu S, Gong L, Fu J, Jin JG, Choi MG, Sohn TS, Lee JH, Bae JM, Kim ST, Park SH, Sohn I, Jung SH, Tan P, Chen R, Hardwick J, Kang WK, Ayers M, Hongyue D, Reinhard C, Loboda A, Kim S, Aggarwal A (2015). Molecular analysis of gastric cancer identifies subtypes associated with distinct clinical outcomes. Nature Medicine.

[ref-5] Deng Q, Liang L, Liu Q, Duan W, Jiang Y, Zhang L (2018). Autophagy is a major mechanism for the dual effects of curcumin on renal cell carcinoma cells. European Journal of Pharmacology.

[ref-6] Falasca L, Torino F, Marconi M, Costantini M, Pompeo V, Sentinelli S, De Salvo L, Patrizio M, Padula C, Gallucci M, Piacentini M, Malorni W (2015). AMBRA1 and SQSTM1 expression pattern in prostate cancer. Apoptosis.

[ref-7] Gil J, Ramsey D, Pawlowski P, Szmida E, Leszczynski P, Bebenek M, Sasiadek MM (2018). The influence of tumor microenvironment on ATG4D gene expression in colorectal cancer patients. Medical Oncology.

[ref-8] Hu D, Jiang L, Luo S, Zhao X, Hu H, Zhao G, Tang W (2020). Development of an autophagy-related gene expression signature for prognosis prediction in prostate cancer patients. Journal of Translational Medicine.

[ref-9] Jassal B, Matthews L, Viteri G, Gong C, Lorente P, Fabregat A, Sidiropoulos K, Cook J, Gillespie M, Haw R, Loney F, May B, Milacic M, Rothfels K, Sevilla C, Shamovsky V, Shorser S, Varusai T, Weiser J, Wu G, Stein L, Hermjakob H, D’Eustachio P (2020). The reactome pathway knowledgebase. Nucleic Acids Research.

[ref-10] Kanehisa M, Furumichi M, Sato Y, Ishiguro-Watanabe M, Tanabe M (2020). KEGG: integrating viruses and cellular organisms. Nucleic Acids Research.

[ref-11] Kutmon M, Van Iersel MP, Bohler A, Kelder T, Nunes N, Pico AR, Evelo CT (2015). PathVisio 3: an extendable pathway analysis toolbox. PLOS Computational Biology.

[ref-12] Lebovitz CB, Robertson AG, Goya R, Jones SJ, Morin RD, Marra MA, Gorski SM (2015). Cross-cancer profiling of molecular alterations within the human autophagy interaction network. Autophagy.

[ref-13] Lee YK, Lee JA (2016). Role of the mammalian ATG8/LC3 family in autophagy: differential and compensatory roles in the spatiotemporal regulation of autophagy. BMB Reports.

[ref-14] Lei Y, Tang L, Hu J, Wang S, Liu Y, Yang M, Zhang J, Tang B (2020). Inhibition of MGMT-mediated autophagy suppression decreases cisplatin chemosensitivity in gastric cancer. Biomedicine and Pharmacotherapy.

[ref-15] Liu PF, Leung CM, Chang YH, Cheng JS, Chen JJ, Weng CJ, Tsai KW, Hsu CJ, Liu YC, Hsu PC, Pan HW, Shu CW (2014). ATG4B promotes colorectal cancer growth independent of autophagic flux. Autophagy.

[ref-16] Liu PF, Tsai KL, Hsu CJ, Tsai WL, Cheng JS, Chang HW, Shiau CW, Goan YG, Tseng HH, Wu CH, Reed JC, Yang LW, Shu CW (2018). Drug repurposing screening identifies tioconazole as an ATG4 inhibitor that suppresses autophagy and sensitizes cancer cells to chemotherapy. Theranostics.

[ref-17] Loncle C, Molejon MI, Lac S, Tellechea JI, Lomberk G, Gramatica L, Zapico MFFernandez, Dusetti N, Urrutia R, Iovanna JL (2016). The pancreatitis-associated protein VMP1, a key regulator of inducible autophagy, promotes Kras(G12D)-mediated pancreatic cancer initiation. Cell Death & Disease.

[ref-18] Mareninova OA, Jia W, Gretler SR, Holthaus CL, Thomas DDH, Pimienta M, Dillon DL, Gukovskaya AS, Gukovsky I, Groblewski GE (2020). Transgenic expression of GFP-LC3 perturbs autophagy in exocrine pancreas and acute pancreatitis responses in mice.1-14. Autophagy.

[ref-19] Mathew R, Karantza-Wadsworth V, White E (2007). Role of autophagy in cancer. Nature Reviews Cancer.

[ref-20] Mizushima N, Yoshimori T, Ohsumi Y (2011). The role of Atg proteins in autophagosome formation. Annual Review of Cell and Developmental Biology.

[ref-21] Nagtegaal ID, Odze RD, Klimstra D, Paradis V, Rugge M, Schirmacher P, Washington KM, Carneiro F, Cree IA (2020). The 2019 WHO classification of tumours of the digestive system. Histopathology.

[ref-22] Nakatogawa H (2013). Two ubiquitin-like conjugation systems that mediate membrane formation during autophagy. Essays in Biochemistry.

[ref-23] Pei L, Kong Y, Shao C, Yue X, Wang Z, Zhang N (2018). Heme oxygenase-1 induction mediates chemoresistance of breast cancer cells to pharmorubicin by promoting autophagy via PI3K/Akt pathway. Journal of Cellular and Molecular Medicine.

[ref-24] Rahman MA, Saha SK, Rahman MS, Uddin MJ, Uddin MS, Pang MG, Rhim H, Cho SG (2020). Molecular insights into therapeutic potential of autophagy modulation by natural products for cancer stem cells. Frontiers in Cell and Developmental Biology.

[ref-25] Rhodes DR, Yu J, Shanker K, Deshpande N, Varambally R, Ghosh D, Barrette T, Pandey A, Chinnaiyan AM (2004). ONCOMINE: a cancer microarray database and integrated data-mining platform. Neoplasia.

[ref-26] Su B, Zhang L, Liu S, Chen X, Zhang W (2019). GABARAPL1 promotes ar+ prostate cancer growth by increasing FL-AR/AR-V transcription activity and nuclear translocation. Frontiers in Oncology.

[ref-27] Sun WL, Wang L, Luo J, Zhu HW, Cai ZW (2018). Ambra1 modulates the sensitivity of breast cancer cells to epirubicin by regulating autophagy via ATG12. Cancer Science.

[ref-28] Tang JY, Hsi E, Huang YC, Hsu NC, Chen YK, Chu PY, Chai CY (2013). ATG9A overexpression is associated with disease recurrence and poor survival in patients with oral squamous cell carcinoma. Virchows Archiv.

[ref-29] Tomczak K, Czerwinska P, Wiznerowicz M (2015). The Cancer Genome Atlas (TCGA): an immeasurable source of knowledge. Contemporary Oncology [Wspolczesna Onkologia].

[ref-30] Van Cutsem E, Sagaert X, Topal B, Haustermans K, Prenen H (2016). Gastric cancer. Lancet.

[ref-31] Wan B, Liu B, Yu G, Huang Y, Lv C (2019). Differentially expressed autophagy-related genes are potential prognostic and diagnostic biomarkers in clear-cell renal cell carcinoma. Aging.

[ref-32] Wu J, Li W, Ning J, Yu W, Rao T, Cheng F (2019). Long noncoding RNA UCA1 targets miR-582-5p and contributes to the progression and drug resistance of bladder cancer cells through ATG7-mediated autophagy inhibition. OncoTargets and Therapy.

[ref-33] Xu R, Liu S, Chen H, Lao L (2016). MicroRNA-30a downregulation contributes to chemoresistance of osteosarcoma cells through activating Beclin-1-mediated autophagy. Oncology Reports.

[ref-34] Yang SW, Ping YF, Jiang YX, Luo X, Zhang X, Bian XW, Yu PW (2016). ATG4A promotes tumor metastasis by inducing the epithelial-mesenchymal transition and stem-like properties in gastric cells. Oncotarget.

[ref-35] Yang Y, Li X, Wang T, Guo Q, Xi T, Zheng L (2020). Emerging agents that target signaling pathways in cancer stem cells. Journal of Hematology & Oncology.

[ref-36] Yusefi AR, Bagheri Lankarani K, Bastani P, Radinmanesh M, Kavosi Z (2018). Risk factors for gastric cancer: a systematic review. Asian Pacific Journal of Cancer Prevention.

